# Multimodal Imaging Techniques Show Differences in Homing Capacity Between Mesenchymal Stromal Cells and Macrophages in Mouse Renal Injury Models

**DOI:** 10.1007/s11307-019-01458-8

**Published:** 2019-12-10

**Authors:** Arthur Taylor, Jack Sharkey, Rachel Harwood, Lauren Scarfe, Michael Barrow, Matthew J. Rosseinsky, Dave J. Adams, Bettina Wilm, Patricia Murray

**Affiliations:** 1 Department of Cellular and Molecular Physiology, University of Liverpool, Liverpool, L69 3BX UK; 2 Centre for Preclinical Imaging, University of Liverpool, Liverpool, UK; 3 Department of Chemistry, University of Liverpool, Liverpool, UK; 4 School of Chemistry, College of Science and Engineering, University of Glasgow, Glasgow, G12 8QQ UK

**Keywords:** Bioluminescence, Magnetic resonance, Kidney disease, MSCs, Macrophages, Stromal cells, Cell tracking, Luciferase

## Abstract

**Purpose:**

The question of whether mesenchymal stromal cells (MSCs) home to injured kidneys remains a contested issue. To try and understand the basis for contradictory findings reported in the literature, our purpose here was to investigate whether MSC homing capacity is influenced by administration route, the type of injury model used, and/or the presence of exogenous macrophages.

**Procedures:**

To assess the viability, whole-body biodistribution, and intra-renal biodistribution of MSCs, we used a multimodal imaging strategy comprising bioluminescence and magnetic resonance imaging. The effect of administration route (venous or arterial) on the ability of MSCs to home to injured renal tissue, and persist there, was assessed in a glomerular injury model (induced by the nephrotoxicant, Adriamycin) and a tubular injury model induced by ischaemia-reperfusion injury (IRI). Exogenous macrophages were used as a positive control because these cells are known to home to injured mouse kidneys. To assess whether the homing capacity of MSCs can be influenced by the presence of exogenous macrophages, we used a dual-bioluminescence strategy that allowed the whole-body biodistribution of the two cell types to be monitored simultaneously in individual animals.

**Results:**

Following intravenous administration, no MSCs were detected in the kidneys, irrespective of whether the mice had been subjected to renal injury. After arterial administration *via* the left cardiac ventricle, MSCs transiently populated the kidneys, but no preferential homing or persistence was observed in injured renal tissue after unilateral IRI. An exception was when MSCs were co-administered with exogenous macrophages; here, we observed some homing of MSCs to the injured kidney.

**Conclusions:**

Our findings strongly suggest that MSCs do not home to injured kidneys.

**Electronic supplementary material:**

The online version of this article (10.1007/s11307-019-01458-8) contains supplementary material, which is available to authorized users.

## Introduction

Numerous studies have shown that mesenchymal stromal cells (MSCs) from various sources, including bone marrow, adipose tissue and the umbilical cord, can improve renal function and ameliorate tissue damage following administration into rodents with kidney injury [1]. However, the mechanisms are ill-defined and it is not clear if the MSCs engraft in injured kidneys or not. Some studies have presented data that suggest MSCs home to injured kidneys, and that renal engraftment is necessary for their therapeutic effects [2, 3], whereas others suggest that MSCs do not engraft and that any therapeutic benefit is likely due to paracrine or endocrine factors [4, 5]. If the former scenario were correct, it would be expected that MSCs administered intravenously (IV) might be less effective in rodent kidney injury models than MSCs administered intra-arterially (*i.e.*, *via* the renal artery, carotid artery, descending aorta or left cardiac ventricle). This is because, following IV administration, most cells become trapped in the lungs due to the pulmonary first-pass effect [6–8], whereas intra-arterial administration delivers more cells to the kidneys [9]. In support of this, a meta-analysis has indicated that the intra-arterial route gives greater benefit in rodents than the IV route [10]. However, biodistribution studies present contradictory findings, with some showing that even after injecting MSCs into the renal artery, they are mainly localised to the lung [11], whereas others show that in animals with renal injury, cells administered arterially are mostly localised in the kidney [12]. These discrepancies could result from a variety of reasons; for instance, different methods being used to induce renal impairment; MSCs being administered at different time points following injury; different tracking methods being used to determine the location of the MSCs. Cell tracking methods that rely solely on identifying MSCs on histological sections using fluorescence microscopy can be particularly problematic due to the fact that the kidney emits intense autofluorescence, which can be increased even further following renal injury [13].

To obtain more accurate information about MSC biodistribution *in vivo* and ascertain how this might be affected by the route of administration, we have previously applied a bi-modal imaging approach comprising bioluminescence imaging (BLI) and magnetic resonance imaging (MRI) to respectively monitor the whole-body and intra-renal biodistribution of mouse MSCs in mice [9]. This was achieved by introducing the firefly luciferase (FLuc) reporter into the MSCs to permit BLI following administration of the substrate, luciferin, and by labelling the MSCs with superparamagnetic iron oxide nanoparticles (SPIONs) so that they could be identified using MRI. In addition to facilitating whole-body imaging, an advantage of FLuc-BLI is that it is an indicator of living cells. A key advantage of MRI is that the spatial resolution is much higher than with BLI, so that it is possible to determine the position of SPION-labelled cells within the kidney [14]. By applying this bi-modal strategy to healthy mice, we found that MSCs administered IV remained trapped in the lungs, but those injected into the left cardiac ventricle could populate the kidneys. Irrespective of administration route, however, most primary MSC types, including human bone marrow-derived MSCs, did not persist in any organ beyond 48 h.

In the current study, using two different mouse injury models where the site of injury is primarily the glomeruli (adriamycin model) or the proximal tubules (ischaemia-reperfusion injury (IRI) model), we have applied this bi-modal imaging strategy to determine if mouse MSCs home to injured kidneys following systemic administration, and whether they persist there. As a positive control cell population, we have used the RAW 264.7 mouse macrophage line which had been reported to ameliorate renal injury in mice [5] and can home to injured tissues [15]. Finally, given that some studies suggest that the therapeutic effects of MSCs are mediated by macrophages [5, 16], we have used a dual-bioluminescence imaging strategy recently developed in our lab that comprises FLuc and a NanoLuc (NLuc)-based bioluminescence resonance energy transfer (BRET) reporter [17], to investigate whether the biodistribution of FLuc^+^ macrophages is affected by co-administration of NLuc^+^ MSCs, and *vice-versa*.

## Methods

### Cell Labelling

The mouse mesenchymal stem/stromal cell (MSC) D1 line was obtained from the American Type Culture Collection (CRL-12424) and RAW 264.7 mouse macrophages were obtained from the European Collection of Authenticated Cell Cultures (#91062702). Both cell types were originally derived from BALB/c mice [18, 19]. Cells were transduced with a lentiviral vectors encoding a bicistronic construct of Firefly Luciferase (FLuc) and ZsGreen under control of the constitutive promoter EF1α or a previously described bioluminescence resonance energy transfer (BRET) reporter [17], also under the control of EF1α. The production and titration of viral particles was performed using established protocols [20]. The cells were transduced with a multiplicity of infection of 5 in the presence of polybrene (8 μg/ml) for the MSCs and without polybrene for the RAW macrophages. At least 90 % of the MSCs expressed the transgenes after transduction whereas macrophages, due to the poor transduction efficiency, were sorted based on ZsGreen fluorescence using an Aria fluorescence-activated cell sorter (BD Biosciences). Both cell lines were maintained in Dulbecco’s Modified Eagle’s medium with 10 % foetal calf serum at 37 °C in a humidified incubator with 5 % CO_2_.

Iron oxide labelling for MR detection was carried out by co-incubation of the cells with the particles for a period of 24 h prior to administration to mice, after which cells were washed and harvested for injection. Cationic SPIONs produced in-house [21, 22] were used for labelling the MSCs (labelling concentration: 25 μg[Fe]/ml; iron content after labelling: ~ 5 pg[Fe]/cell) whereas macrophages were labelled with ferumoxytol (AMAG pharmaceuticals, labelling concentration: 20 μg[Fe]/ml, iron content after labelling: ~ 6.5 pg[Fe]/cell). The use of different SPIONs is required because each cell type responds differently to such materials, *e.g*., ferumoxytol alone does not effectively label MSCs [23], but do label RAW macrophages without affecting their morphology or viability [24]. In all experiments, cells were trypsinised, pelleted, resuspended in ice-cold phosphate buffered saline (PBS) and kept on ice until injection. The cell suspension (100 μl) was administered to mice intravenously *via* the tail vein or intracardially (IC) *via* the left ventricle of the heart *via* ultrasound guidance (Prospect system, S-Sharp, Taiwan) [9].

### Models of Kidney Injury

BALB/c mice (Charles River, UK) were housed in individually ventilated cages under a 12-h light/dark cycle, with *ad libitum* access to standard food and water. All animal experiments were performed under a licence granted under the UK Animals (Scientific Procedures) Act 1986 and were approved by the University of Liverpool ethics committee. ARRIVE guidelines were followed to report animal experiments. This mouse strain is expected to accept the two cell lines used in this study, irrespective of animal’s immune status.

Adriamycin nephropathy was induced in female BALB/c SCID mice by injecting adriamycin (ADR, doxorubicin hydrochloride, Tocris, UK) IV at 6.3 mg/kg body weight (BW) in 0.9 % saline. Cells were administered 14 days post ADR, when renal impairment is observed [25]. For IRI, male mice were anaesthetised with isoflurane and a flank incision was made for unilateral clamping of the renal pedicle for 40 min, using an atraumatic vascular clamp. Subsequently, the vascular clamp was removed and restoration of renal blood flow confirmed visually prior to repair of muscle and skin layers. In this model, renal impairment is observed 24 h post-clamping [26] and cells were administered at this time point.

### Imaging

For bioluminescence imaging, a subcutaneous injection of luciferin (150 mg/kg body weight, Promega, UK) was administered to mice under anaesthesia, which were imaged 15 min later in a bioluminescence imager (IVIS Spectrum, Perkin Elmer, UK). Imaging data were normalised to the acquisition conditions and expressed as radiance (photons/s/cm^2^/sr (p/s/cm^2^/sr)). For *ex vivo* BLI, mice were culled 10 min post administration of luciferin, after which organs were harvested and immediately imaged. Bioluminescence signals of whole animals or individual organs *ex vivo* were quantified by drawing regions of interest (ROIs) from which the total flux (photons/second) was obtained. For simultaneous tracking of MSCs and RAWs in the same animal, MSCs were transduced with a BRET reporter and imaged *in vivo* and *ex vivo* as previously reported [17]. In brief, the imaging protocol involves the tail vein cannulation of the mice, injection of the substrates (furimazine or luciferin) IV and sequential acquisition of data. The BRET signal from the MSCs is obtained immediately after furimazine injection and because the half-life of this substrate is very short, the signal is cleared within approximately 10 min. This allows subsequent injection of luciferin and collection of Fluc signal from the RAW macrophages in the same imaging session, without crosstalk between the different reporter systems. *Ex vivo* imaging involves a similar method, but the firefly luciferase signal is collected before the BRET signal [17].

MR data was obtained with a Bruker Avance III console interfaced to a 9.4 T magnet system (Bruker Biospec 94/20 USR) and a 4-channel receive-only abdominal surface coil in combination with a 72-mm resonator. Mice were imaged at baseline (before injury) and at multiple time points post administration of SPION-labelled cells. A gated Fast Low-Angle Shot (FLASH) T_2_* sequence was used with the following acquisition parameters: TE 5.5 ms, TR 262.5 ms, flip angle 20 °, matrix size 386 × 386 pixels, field of view 35 × 35 mm, slice thickness 0.5 mm, number of slices 20, averages 3, acquisition time 5 min, 35 s. T_2_^*^ relaxation times were obtained from a T_2_^*^ map by drawing ROIs around the cortex of the kidney or a region of the liver as previously described [9]. The T_2_^*^ maps were obtained with a multigradient echo (MGE) sequence with 8 echoes starting at 4.5 ms with 4.5 ms increments and TR 900 ms, flip angle 50 °, matrix size 256 × 256 pixels, field of view 35 × 35 mm, slice thickness 0.5 mm, number of slices 20, averages 2, acquisition time 5 min, 45 s. For *postmortem* imaging of kidneys, the organs were fixed in formaldehyde, embedded in agarose and imaged with a FLASH T_2_^*^ sequence with following acquisition parameters: TE 6.3 ms, TR 1300 ms, flip angle 20 °, matrix size 400 × 400 pixels, field of view 17 × 17 mm, slice thickness 0.2 mm, number of slices 70, averages 24, acquisition time 3 h, 20 min.

## Results

### A Combination of MRI and BLI Allows the *in vivo* Imaging of MSC Delivery to the Kidneys, but Does Not Provide Evidence of Preferential Persistence or Homing to the Injured Kidney

**Fig. 1.**
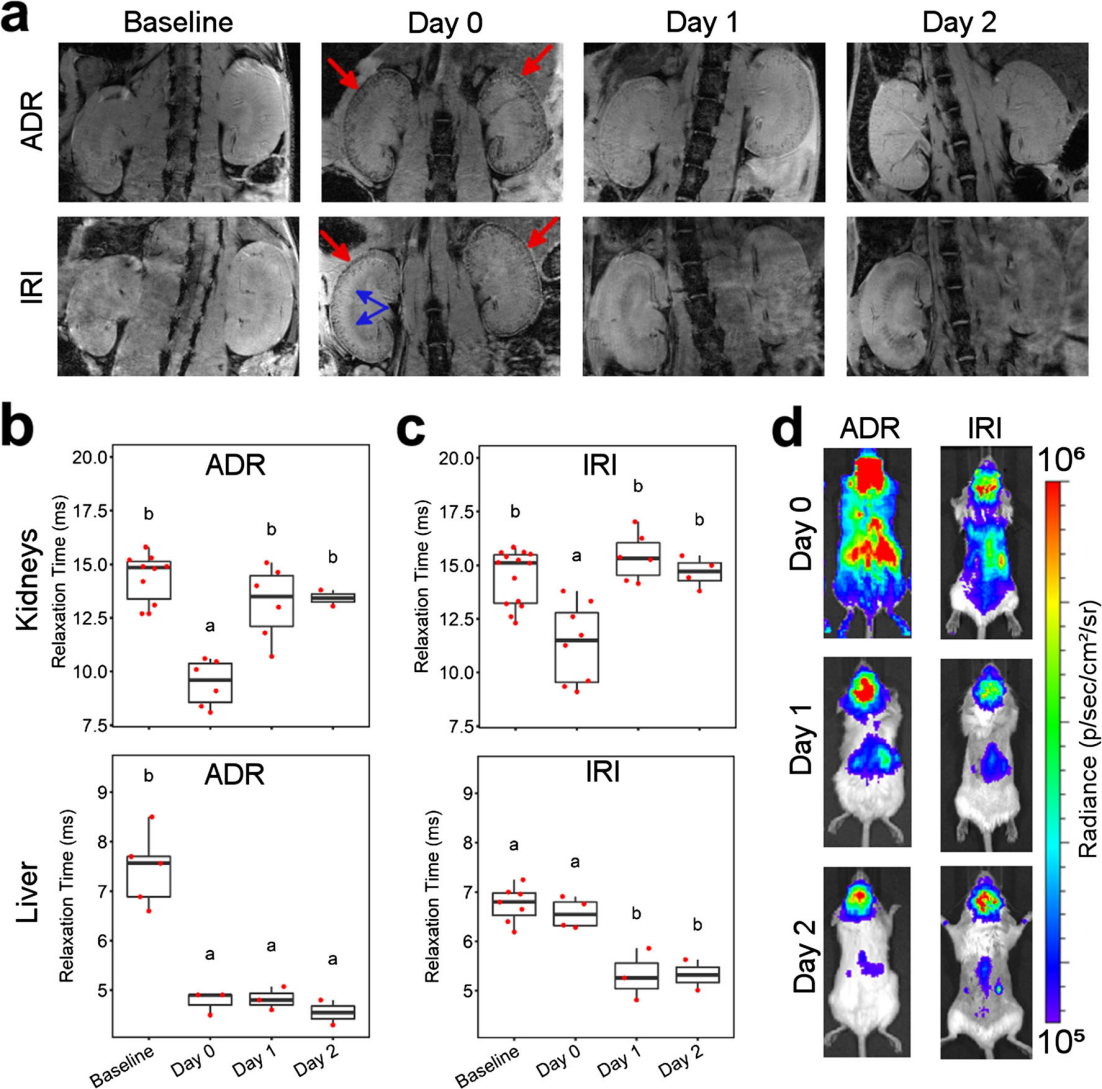
*In vivo* MR/BL imaging provides evidence of MSC delivery to the kidneys, but no clear differences in the presence of a kidney injury. **a** MRI of the kidneys at baseline (prior to injury) and up to 2 days post intracardiac administration of 10^6^ cells to SCID mice with an adriamycin or ischaemia-reperfusion renal injury. On the cell administration day (day 0), hypointense contrast was seen in the cortices of the kidneys (red arrows), indicating cell delivery. The contrast is lost in subsequent days. In the IRI model, hypointense contrast is also seen in the medulla of the injured kidney (blue arrows). **b** T_2_^*^ relaxation time of the kidneys’ cortices and liver in the ADR model. **c** T_2_^*^ relaxation time of the kidneys and liver in the IRI model. Time points that do not share the same letters are significantly different from one another, *p* < 0.05 (Tukey’s *post hoc* test). *p* values for all comparisons are shown in SI Table 1. **d** BLI on cell administration day (day 0) shows whole-body distribution of cells, including the kidneys but the signal is progressively lost in subsequent days, suggesting cell death

**Fig. 2.**
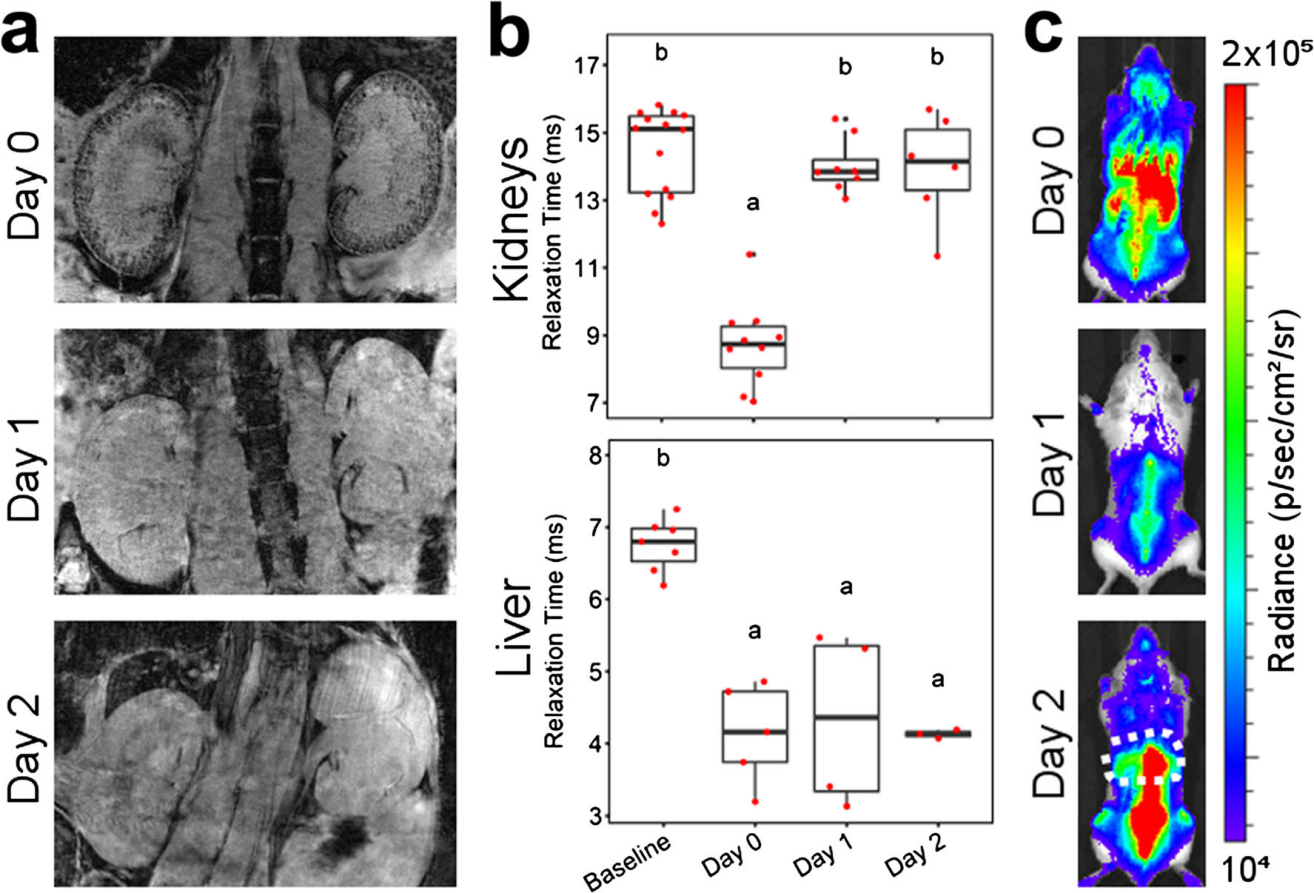
*In vivo* MR/BL imaging provides evidence of macrophage delivery to the kidneys, but no clear differences between injured (left) and healthy (right) kidney. **a** MRI of the kidneys at baseline and up to 2 days post administration of 5 × 10^6^ cells to SCID mice with an ischemia/reperfusion injury. On the cell administration day (day 0), hypointense contrast is seen in the cortices of the kidneys, indicating cell delivery. The contrast is lost in subsequent days. **b** T_2_^*^ relaxation time of the kidney cortices and livers. Time points that do not share the same letters are significantly different from one another, *p* < 0.05 (Tukey’s *post hoc* test). *p* values for all comparisons are shown in SI Table 2. **c** BLI on cell administration day (day 0) shows whole-body distribution of cells, including the kidneys but the signal changes progressively in subsequent days, and by day 2, the signal is predominately found in the liver (dashed area) and spine

We applied an imaging protocol involving the double labelling of MSCs with SPIONs and FLuc to allow their imaging *via* MRI and BLI, respectively. In MRI, the SPION label creates local magnetic field inhomogeneities in the areas where cells are delivered to, leading to decay of transverse magnetisation, which is observed as hypointense (dark) contrast in T_2_^*^-weighted imaging. This is usually used to image a specific organ or body region. BLI, on the other hand allows assessment of the whole-body distribution of cells. SPION^+^FLuc^+^ MSCs were administered into the left cardiac ventricle of mice with adriamycin-induced injury or IRI. Irrespective of the injury model, MR imaging performed within a few hours of cell administration showed hypointense contrast in the renal cortices (Fig. 1a). This confirmed that administration *via* the arterial route leads to successful delivery of the cells to the kidneys. Hypointense contrast was additionally observed in the medulla of kidneys following IRI (Fig. 1a, blue arrows); however, this phenomenon was also observed in the absence of administered cells (see Fig. 1 in Electronic Supplementary Material (ESM)), suggesting a surgery-specific effect. The contrast in the renal cortices was progressively lost in the subsequent days, indicating that the cells were cleared from the kidneys. Quantification of the T_2_^*^ relaxation time, the time constant that describes the decay of transverse magnetisation, revealed a statistically significant reduction on the administration day but recovery to baseline values in the following days (Fig. 1b, c). For mice that underwent IRI, we compared the relaxation times of injured kidney with that of the uninjured contralateral kidney but saw no differences between the two (see Fig. 2a in ESM). In the liver of IRI mice, the T_2_^*^ relaxation time dropped progressively throughout the experimental period to values that were significantly different from baseline by days 1 and 2, whereas the T_2_^*^ relaxation time was significantly lower than baseline on all days after cell administration in the ADR model (Fig. 1b, c). This suggests that SPIONs are transported from the kidneys to the liver in the days subsequent to cell administration. BLI revealed whole-body distribution of MSCs on the administration day, as anticipated from injection *via* the arterial route. A progressive loss of signal intensity was seen in the days following cell administration, suggesting cell death. By day 2, only a weak signal was detected in the kidneys, which is consistent with the loss of MR contrast. Taken together, these data suggest loss of MSCs from the kidneys *via* progressive cell death, following which, the SPION label is transported to the liver, most likely by the host’s reticuloendothelial system.

### Similarly to MSCs, RAW Macrophages Are Short-Lived in the Kidneys but Populate the Liver

**Fig. 3.**
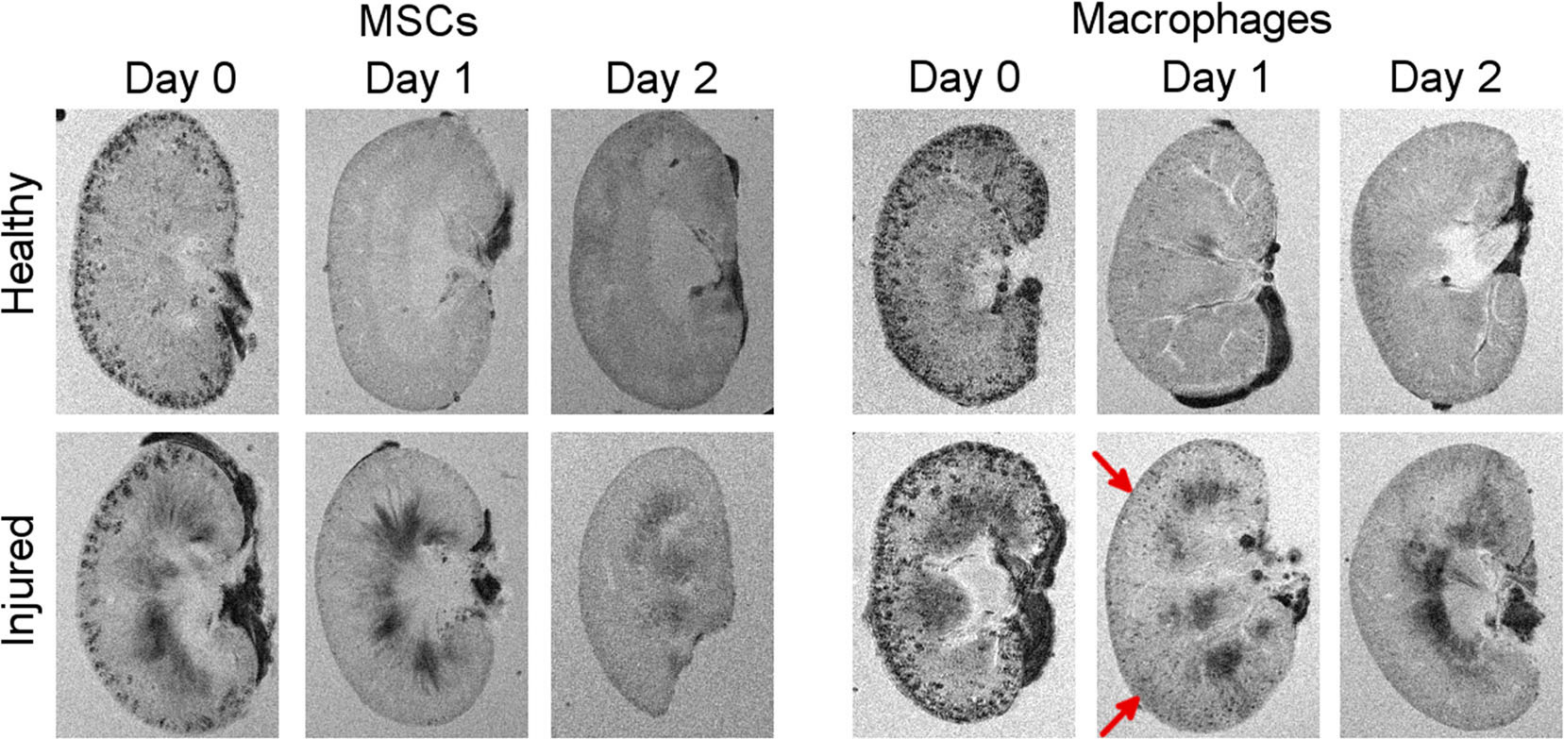
*Ex vivo* high-resolution MRI supports the findings observed *in vivo* (Figs. 1 and 2). On day 1, more hypointense areas appear to be present in the injured kidneys of SCID mice that underwent IRI and received macrophages (red arrows)

**Fig. 4.**
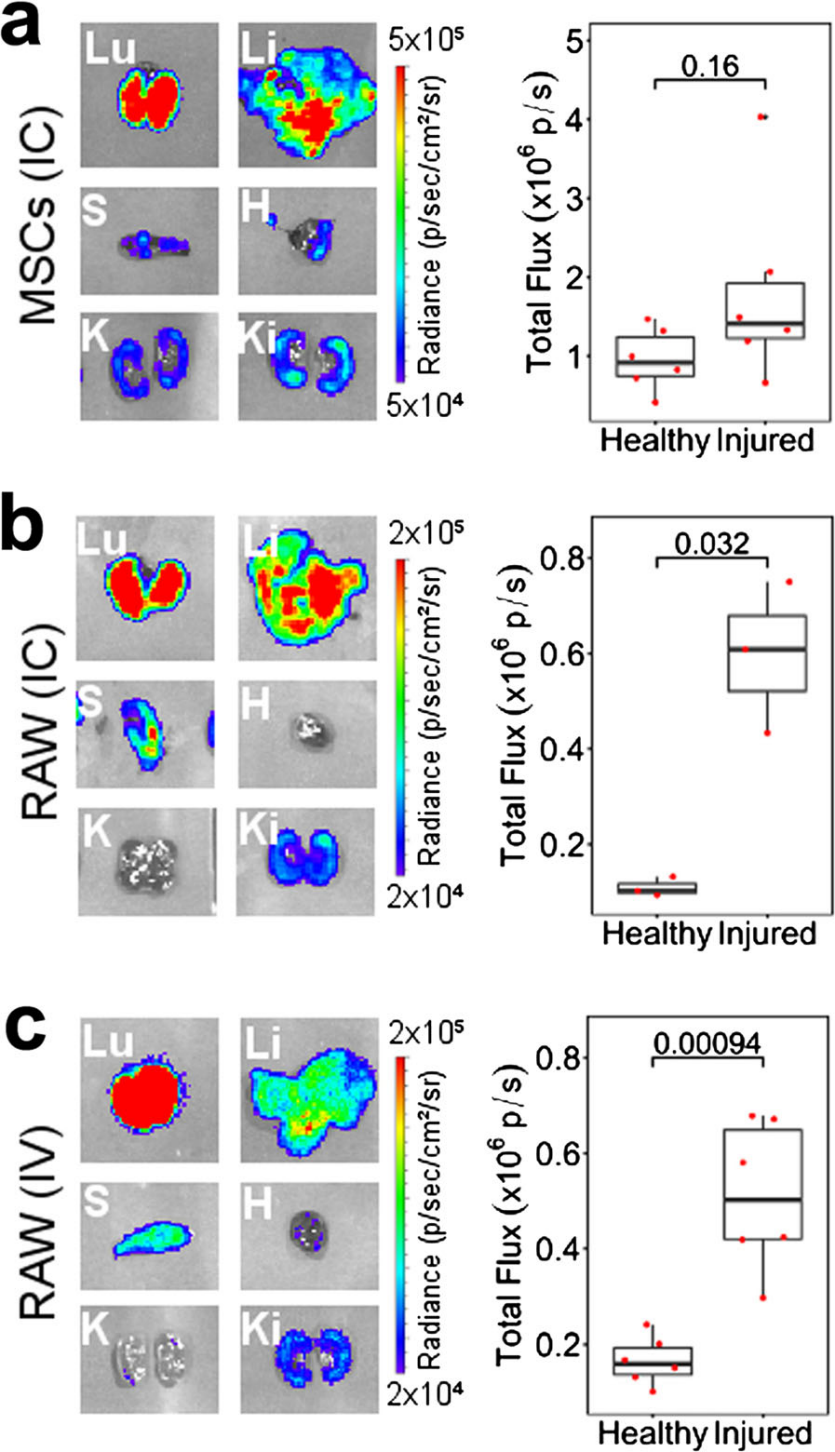
*Ex vivo* BLI is more sensitive and suggests preferential survival or retention of cells in the injured kidney of mice that underwent IRI. **a** 10^6^ MSCs administered IC, **b** 5 × 10^6^ RAWs administered IC, and **c** 10^7^ RAWs administered IV. Left: Representative BLI of individual organs on day 1 post cell administration. Organs from the same animal were imaged at the same time and under the same acquisition conditions. Lu, lungs; Li, liver; S, spleen; H, heart; K, healthy kidney; Ki, injured kidney. Right: Quantification of the total flux from each of the kidneys. MSC IC, *n* = 6; RAW IC, *n* = 3; RAW IV, *n* = 6

Having observed no persistence of MSCs in the kidneys, we investigated whether macrophages have a different fate. From here, our study focuses on the unilateral IRI model given the presence of an internal control kidney in each animal. A total of 5 × 10^6^ cells were administered, based on a dose-finding study that showed that these cells are well tolerated when injected *via* the left ventricle of the heart [9]. MR imaging revealed a similar renal distribution as observed with the MSCs, with strong negative contrast in the cortex on the administration day and progressive loss on the following days (Fig. 2a). Indeed, the changes in T_2_^*^ relaxation times in the renal cortices and liver follow the same behaviour as we observed for the MSCs (Fig. 2b), and as before, no differences were seen when comparing the injured kidney with the control kidney (see Fig. 2b in ESM). However, when animals were analysed individually, we noticed that some appeared to display a greater negative contrast in the injured kidney on the administration day (see Fig. 3 in ESM). BLI imaging displayed a different distribution to that seen with the MSCs, with a moderate loss of signal on day 1, but an increase in signal in the spine and liver by day 2 suggesting that the cells had populated these organs (Fig. 2c). This implies that for this cell type, the reduction in T_2_^*^ relaxation time in the liver was probably a combination of two components: (i) SPION debris from RAW macrophages that have died after administration and (ii) viable SPION^+^ FLuc^+^ RAW macrophages that home to the liver. The differences in cell fate between the two cell types are also evident when mice are imaged ventrally (see Fig. 4 in ESM), with a marked signal originating from the liver of mice that receive macrophages but not from those that receive MSCs.

To confirm the MR data obtained *in vivo*, we scanned the kidneys *postmortem* at a higher resolution. *Ex vivo* imaging of the kidneys of mice that received MSCs exhibited the same features as observed *in vivo*: (i) hypointense contrast in the renal cortex on day 0, which was lost in subsequent days; (ii) no major differences between the distribution of cells in the cortex of healthy and injured kidneys; and (iii) a darkening of the medulla of the injured kidney (Fig. 3). Imaging of the kidneys of animals that received macrophages displayed a similar pattern, but the contrast in the cortex was stronger which is likely a consequence of the higher injection dose (5 × 10^6^ RAWs *vs* 10^6^ MSCs), meaning that more cells are present in the kidneys on the administration day. Interestingly, a careful examination of the images revealed that more hypointense spots were present in the injured kidney, particularly on day 1, when compared with the healthy kidney (Fig. 3, red arrows). Taken together, the *in vivo* and *ex vivo* MR imaging of macrophages suggest a greater accumulation or persistence of cells in the injured kidney, but this could not be unambiguously demonstrated in a quantitative manner using this imaging modality.

### BLI *ex vivo* Provides Improved Sensitivity and Reveals Distinct Behaviour Between Macrophages and MSCs

To quantitatively determine whether macrophages do preferentially persist in the injured kidney as suggested by the MR data, mice were culled 24 h post cell administration for *ex vivo* imaging of the organs *via* bioluminescence. Imaging of the lungs, liver, spleen, heart and kidneys revealed that cells were present in all major organs at this time point with lungs and liver displaying the strongest signal intensity (Fig. 4). Analysis of the signal intensity in each of the kidneys showed an increase in the mean signal in the injured kidneys when mice received the MSCs IC, but this was not statistically significant (*p* = 0.16, Fig. 4a). Mice that received the macrophages IC, on the other hand, displayed a statistically significant difference between kidneys, with the injured having a greater bioluminescence intensity (*p* = 0.032, Fig. 4b). Because it is known that RAW macrophages are able to bypass the lung vasculature when administered IV [9], we assessed whether this route of administration can also lead to cells accumulating preferentially in the injured kidney. Administration of 10^7^ cells *via* this route confirmed their ability to extravasate the lungs, with cells also populating the liver, spleen and kidneys (Fig. 4c). A stronger bioluminescence intensity was detected in the injured kidneys (*p* = < 0.001) suggesting a homing effect where these cells actively migrate to the site of ischaemic injury. Of note, our experimental setup only allowed us to detect those differences when imaging the organs *ex vivo*. Our attempts to quantify the signal emanating from the kidneys *in vivo* were unsuccessful due to the presence of cells in other organs, the surgical scar overlying the injured kidney and the low spatial resolution of BLI (ESM Fig. 5).

**Fig. 5.**
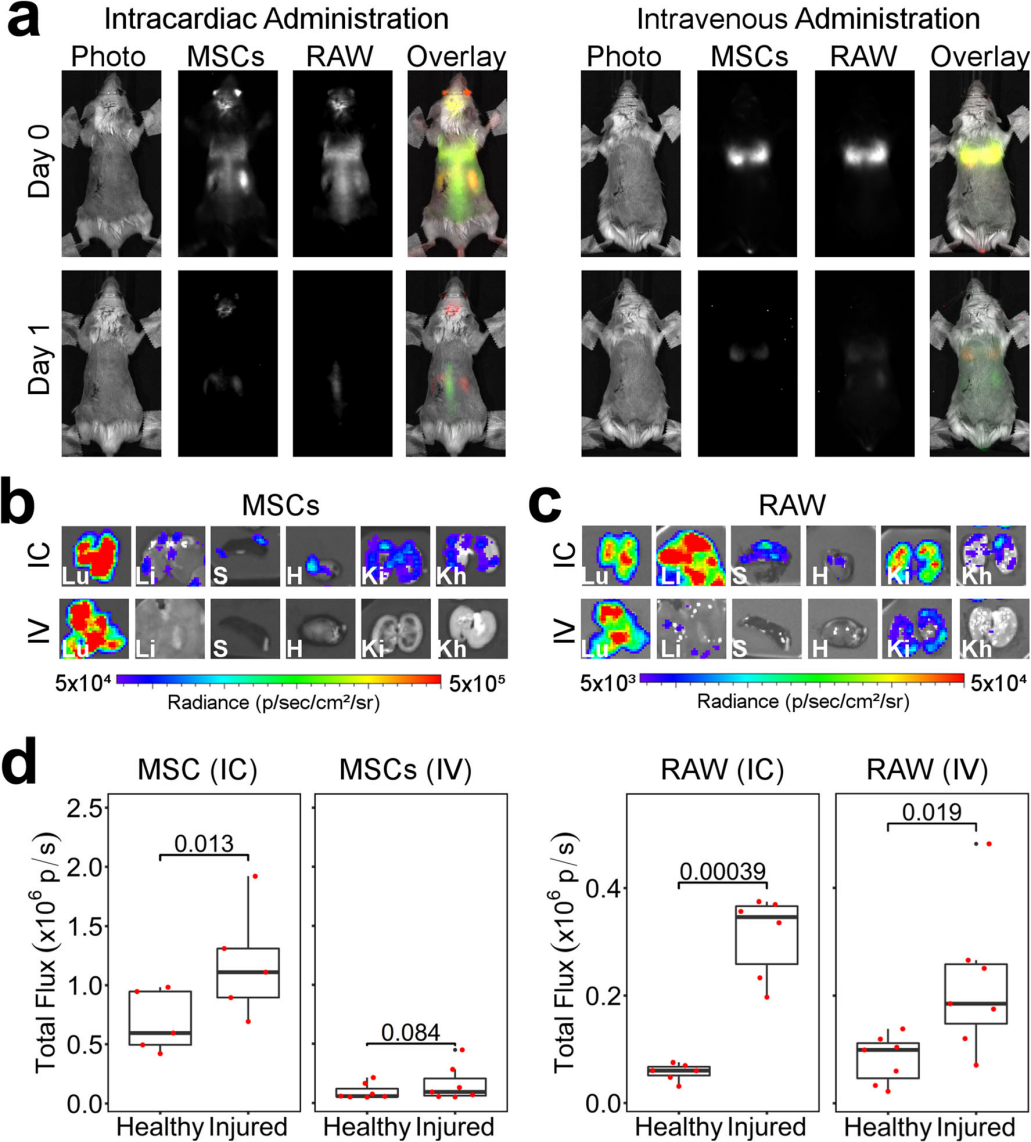
Simultaneous *in vivo* tracking of MSCs and RAW macrophages co-administered to SCID mice that underwent IRI. **a** BLI signal as obtained from MSCs (BRET reporter) or macrophages (Fluc) without any colour scaling, and an overlay with pseudo-colours (red: MSCs, green: macrophages). The same images, displayed with a “rainbow” scale for each of the conditions, are shown in the Fig. 6 in ESM. **b**, **c** Representative images of organs imaged *ex vivo* on day 1 post administration (b, MSCs; c, RAWs). **d** Quantification of the total flux from each of the *ex vivo* kidneys. IC administration *n* = 6, IV administration *n* = 7

### Multiplex Imaging Allows the Tracking of each Cell Independently and Suggests no Crosstalk Between RAW Macrophages and MSCs

Having determined differences in the homing and persistence of MSCs and RAW macrophages in mice with IRI, we next sought to identify whether the biodistribution of the MSCs could be influenced by the presence of exogenous macrophages. For this, we co-injected MSCs and RAW macrophages into the same mice and applied a method to track them individually, using a combination of NanoLuc-based BRET reporter and FLuc. Shortly following IC administration, both cell types showed a similar whole-body distribution, with good co-localisation of the MSC and macrophage signal on day 0 (Fig. 5a). On day 1, the signal weakened, in agreement with the data in Figs. 1 and 2, with some MSCs still present in the abdominal area, including the kidneys and brain, whereas the macrophage signal was most intense in the spine. A different scenario emerged when the cells were administered IV, with both types of cells being found exclusively in the lungs on the day of administration. A strong reduction in signal intensity was observed by day 1, with MSCs still located in the lungs, whereas macrophages were found not only in the lungs, but also in the abdomen.

*Ex vivo* imaging of organs on day 1 showed similar results to those obtained when the cells were administered individually. After IC administration, MSCs were found in most organs including the kidneys, with a stronger signal intensity in the injured kidney (Fig. 5b). In contrast to the data shown in Fig. 4a, we saw a statistically significant difference between the kidneys in this experimental setup. We have previously shown that MSCs expressing this BRET reporter have a light output much greater than that of cells expressing FLuc [17] and it is thus possible that these results reflect a greater sensitivity of the reporter, allowing us to detect a statistically significant difference which was not observed with FLuc. However, it is also possible that the increased numbers of RAW macrophages in the injured kidney may restrict blood flow through the glomerular capillaries, causing a transient accumulation of MSCs. Following IV administration, no MSCs were detected in any organ apart from the lungs (Fig. 5b). RAW macrophages, on the other hand, produced a stronger signal in the injured kidney that was statistically significant irrespective of the administration route, demonstrating that their homing is not affected by co-administration of MSCs.

## Discussion

MSCs are efficacious in various preclinical models of renal injury [10], but whether their therapeutic effects are dependent on their ability to populate the kidneys is a contested issue; for instance, some reports have suggested that efficacy is improved with enhanced renal homing of MSCs [2], while others suggest that improvements in renal health occur in the absence of homing [4, 5]. To determine whether MSCs home to injured kidneys or not, we used multimodal imaging to assess MSC biodistribution following IV and IC administration in two different mouse models of renal injury. In contrast to macrophages, which served as a positive control, we found little evidence of any MSC renal homing capacity.

We have previously shown that stem cell administration *via* the intracardiac route prevents the well-known pulmonary first-pass effect, allowing cells to reach the kidneys [9]. However, in this previous study only healthy mice were used. Here, we sought to assess whether the presence of a glomerular (Adriamycin) or tubular (IRI) injury affects the persistence and survival of the cells in this organ. In the former model, both kidneys are injured, requiring comparison with a control group. Comparison with our previous data, which involved the same mouse strain, same MSC line and the same labelling/imaging strategy [9] reveals that survival and persistence are not affected by renal injury, with the great majority of the cells dying or being cleared from the kidneys in the days subsequent to their administration. When compared with the ADR model, the unilateral ischemia/reperfusion model provides the advantage of an internal control within the same animal. The behaviour of the cells in the IRI model was similar to that observed with healthy or adriamycin-injured animals; that is, an initial accumulation was observed in the renal cortices followed by cell death and/or rapid clearance. A similar dynamic was observed when injecting macrophages. It is important to note that the ADR is a model of glomerular injury, while IRI predominantly damages the proximal tubules. Nevertheless, MRI signal after IC administration was seen predominantly in the cortices, but not in the renal tubules, irrespective of the injury model. We have previously shown that when administered IC, cells that reach the kidney are found predominantly in the glomeruli [25], which are highly vascularised. These observations indicate that although the IC route results in good delivery of MSCs to the kidneys, their distribution in this organ likely relates to vascular entrapment rather than to active homing to the site of injury.

Bioluminescence imaging the organs *ex vivo* offers greater sensitivity because there is much less signal attenuation in the absence of surrounding tissue. On the first day following macrophage administration, a difference in the signal intensity between the injured and control kidney in the IRI model was observed, which has two important implications.

The first concerns the sensitivity of the technologies used here. The combination of MRI and BLI *in vivo* provided important information to whether cells reached the kidneys as well as their intra-renal distribution and long-term survival, but was not sufficiently robust to allow us to detect subtle differences in signal between the two kidneys. Although in the case of macrophages, qualitative differences were seen between the kidneys of individual animals *via* MRI *in vivo*, these were not reflected when the mean relaxation time of whole groups was compared. This shows the need to confirm *in vivo* data *postmortem*.

The second implication concerns the biological response of administered cells to the renal injury. Administration of MSC and RAWs *via* the IC route led to a stronger BLI signal in the injured kidneys on day 1, implying the presence of a greater number of cells. We have not yet established the specific mechanisms on which the stronger signals in the injured kidneys are based, but important questions arise: Are the administered cells attracted to the site of injury due to local chemokine release? Is the phenomenon based on physical entrapment due to underlying changes in the structure of the kidney as a result of the ischaemic injury? The observation that IV injected macrophages also produce a stronger signal in the injured kidney suggests that at least for this cell type, chemoattraction likely takes place. Indeed, it is well recognized that chemokine-mediated infiltration of macrophages takes place in ischaemic acute kidney injury [27]. This has additional implications when their action as a cell therapy is taken into consideration, as it suggests that both the venous and arterial routes are effective in delivering macrophages to the site of injury. Importantly, the same has not been seen with MSCs. Although previous studies have suggested that MSCs bypass the lungs and reach the kidneys after IV administration, most of those studies used imaging methods that are prone to false positives, *e.g.,* (i) the use of lipophilic dyes [28] that can be transferred to host cells [29] or (ii) reliance on histological sections, which can give false positive results due to the increased levels of autofluorescence in injured kidneys [13, 29].

Our imaging with reporter genes that are specific to viable cells provides clear, unambiguous evidence that IV administered MSCs do not bypass the lungs in two mouse models of renal injury, and is in agreement with our previous data using healthy animals [25]. Thus, any positive effects on tissue regeneration and/or repair seen after IV administration of MSCs are likely related to mechanisms that do not involve the cells migrating and integrating with the renal tissue. Further, this study reinforces the utility of combining multiple reporter gene systems to individually track the dynamics of cell distribution and persistence in different organs not only *in vivo* but also *postmortem* in excised tissues.

## Conclusions

By applying an imaging strategy combining BLI and MRI, we have been able to determine that the delivery of cell therapies to the kidneys is dependent on cell type and route of administration in murine models of renal injury. MSCs do not home to the kidneys and are unable to bypass the lungs when administered intravenously. Macrophages, on the other hand, have a capacity to home and accumulate preferentially in the injured kidneys, whether they are administered *via* the venous or arterial circulation. Multiplex BLI enabled us to track the biodistribution and persistence of each of these different cell types individually in the same animal, revealing that their fate is independent of one another.

## Electronic supplementary material

ESM 1 (PDF 1322 kb)
