# Supplementary material for: Multimodal Imaging Techniques Show Differences in Homing Capacity Between Mesenchymal Stromal Cells and Macrophages in Mouse Renal Injury Models
Source: Mol Imaging Biol. 2019 Dec 10;22(4):904–13. doi: 10.1007/s11307-019-01458-8 (PMC7343735; doi:10.1007/s11307-019-01458-8)
Supplement: Supplementary file 1 — (PDF 1322 kb) [file 11307_2019_1458_MOESM1_ESM.pdf]

Electronic Supplementary Material

Multimodal Imaging Techniques Show Differences in Homing Capacity Between Mesenchymal Stromal Cells and Macrophages in Mouse Renal Injury Models

Journal: Molecular Imaging and Biology

Arthur Taylor[1,2,§], Jack Sharkey[1,2,§], Rachel Harwood[1,2,§], Lauren Scarfe[1,2,§], Michael Barrow[3], Matthew J. Rosseinsky[3], Dave J. Adams[4], Bettina Wilm[1,2,*], Patricia Murray[1,2,*]

1. Department of Cellular and Molecular Physiology, University of Liverpool, Liverpool, UK.

2. Centre for Preclinical Imaging, University of Liverpool, Liverpool, UK.

3. Department of Chemistry, University of Liverpool, Liverpool, U.K.

4. School of Chemistry, College of Science and Engineering, University of Glasgow, Glasgow, U.K.

§ Authors contributed equally, * Joint senior/corresponding authors

Correspondence information for corresponding authors:

Patricia Murray, PhD, Department of Cellular and Molecular Physiology, Institute of Translational Medicine, University of Liverpool, Liverpool L69 3BX, UK; Tel: +44-151-7945450, email: p.a.murray@liverpool.ac.uk

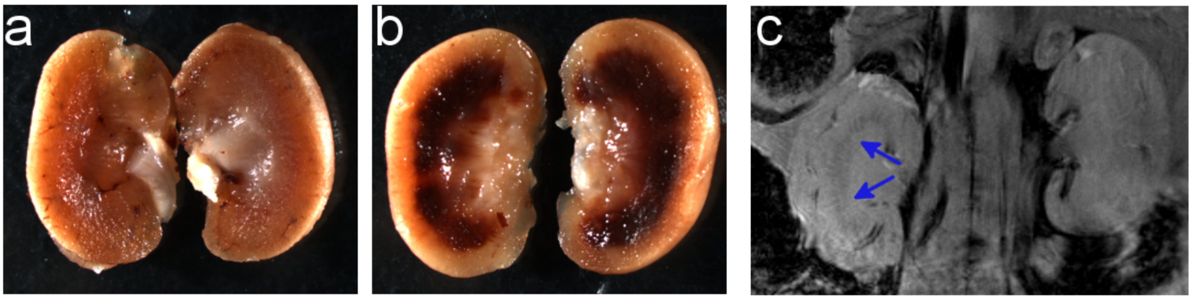

ESM Fig 1. Aspect of an **(a)** healthy and **(b)** injured kidney of the same animal, 1 day post IRI. Blood coagulation is seen in the medulla of the injured kidney. **(c)** MRI of the kidneys of a mouse 1 day post IRI. Although this mouse received no SPION-labelled cells, hypointense contrast in the medulla is observed (blue arrows) as a consequence of blood coagulation.

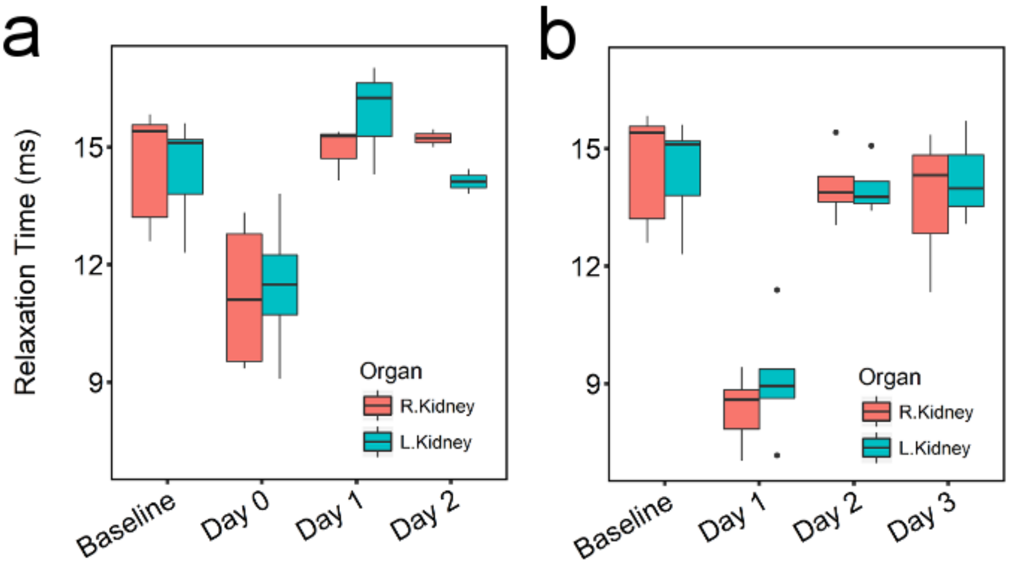

ESM Fig 2. Comparison of the cortical relaxation time in injured (right) *vs.* uninjured (left) kidney for mice that received either (a) MSCs or (b) macrophages. No statistically significant differences are seen between kidneys. p-values are shown in ESM Table 3.

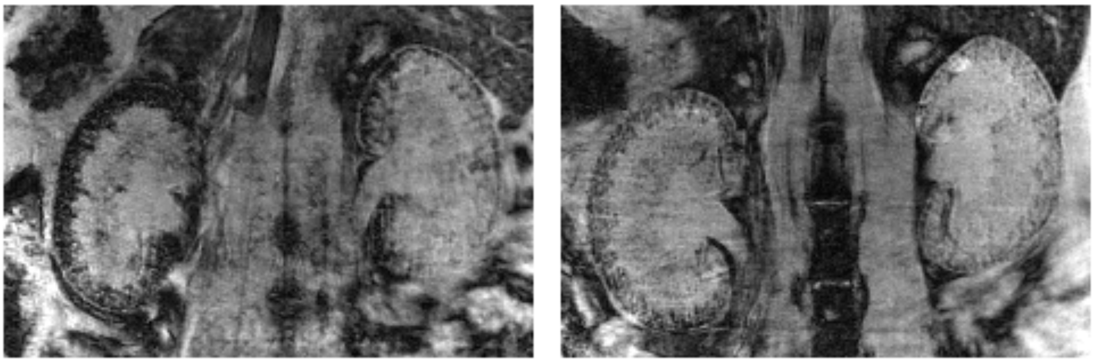

ESM Fig 3. Examples of MR imaging of mice that, qualitatively, appeared to display a stronger hypointense signal in the injured (right) kidney immediately after administration of RAW macrophages.

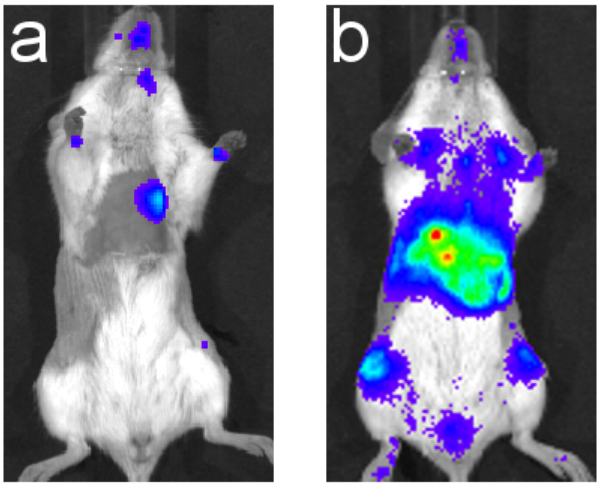

ESM Fig 4. Representative ventral images 48h post administration of (a) MSCs or (b) macrophages. While most MSCs die during the course of the experiment, macrophages accumulate and proliferate in the livers. BLI scale: (a) 10^5 to 10^6 photons/sec/cm2/str, (b) 10^4 to $2x10^5$ photons/sec/cm2/str.

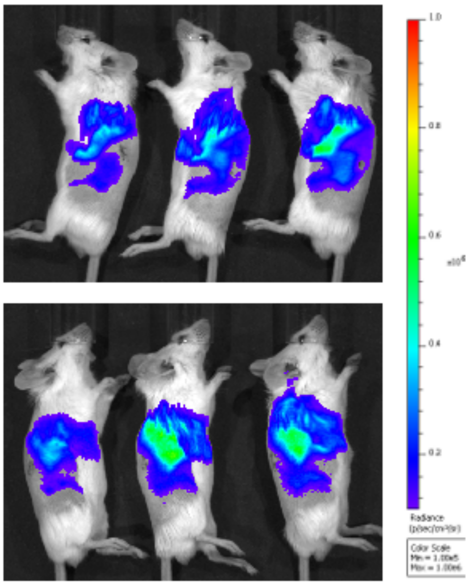

ESM Fig 5. Lateral imaging of mice 24 h post IV administration of macrophages. Discriminating and quantifying the specific signal contribution of the kidneys in *in vivo* imaging is difficult because cells are also present in other organs such as lungs, liver and spleen. BLI scale: 10^5 to 10^6 photons/sec/cm^2/str.

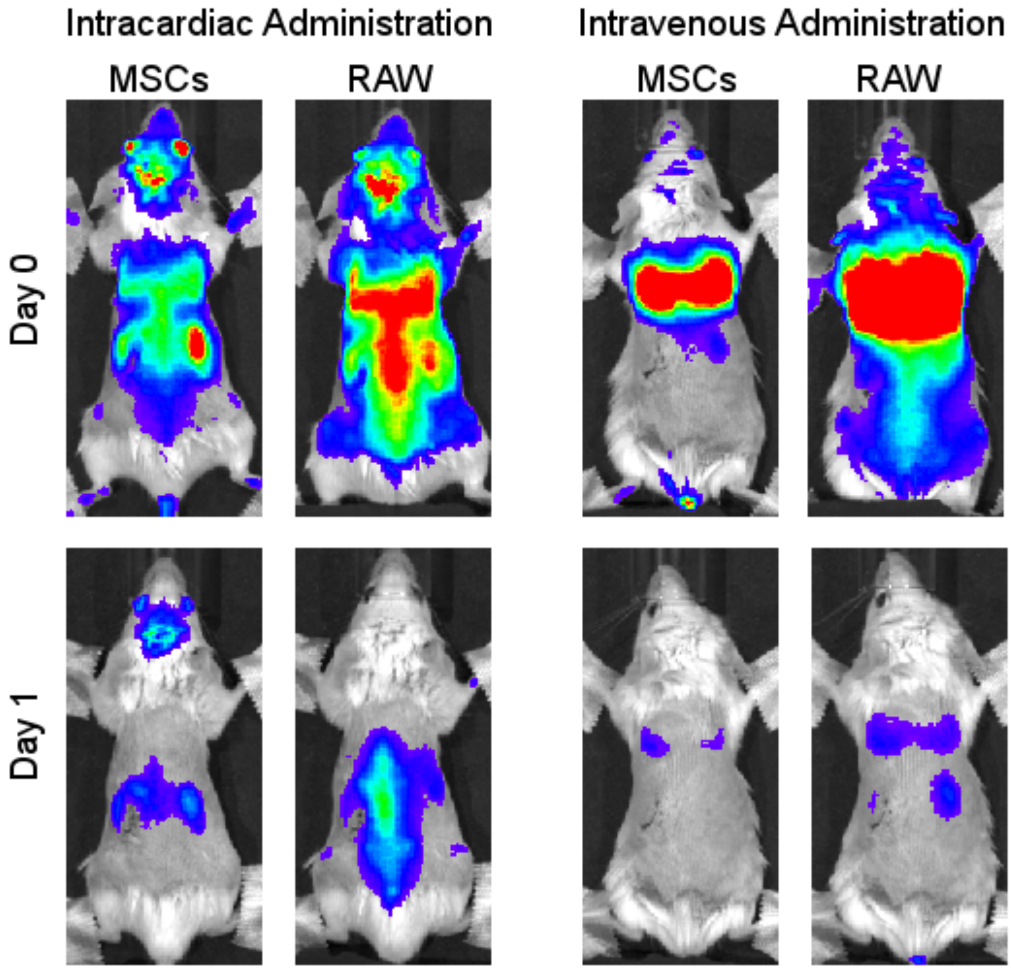

ESM Fig 6. *In vivo* data from Fig. 5 displayed with a "rainbow" colour scale. Radiance scale: MSCs $1x10^5$ to $1x10^6$ photons/sec/cm2/str, Macrophages: $2x10^5$ to $2x10^5$ photons/sec/cm2/str.

ESM Table 1. p-values from post-hoc Tukey's HSD test for comparisons of relaxation time of kidneys and livers up to 2 d post MSC administration. ANOVA in the ADR model: livers p= $8.11x10^{-5}$, kidneys p = $3.99x10^{-6}$, ANOVA in the IRI model, livers p = $3x10^{-4}$, kidneys p = $1.8x10^{-5}$.

**Comparison**	**ADR model**		**IRI Model**	
	**Liver**	**Kidneys**	**Liver**	**Kidneys**
Day 0-Baseline	0.0003358	0.0000018	0.8823047	0.0001091
Day 1-Baseline	0.0003947	0.3164446	0.0009036	0.5049237
Day 2-Baseline	0.0005213	0.7774173	0.0032296	0.9922368
Day 1-Day 0	0.9991511	0.0002832	0.0057814	0.0000421

Day 2-Day 0	0.9689246	0.0052496	0.0144329	0.0024198
Day 2-Day 1	0.9409844	0.9963331	0.9999917	0.8448341

ESM Table 2. p-values from post-hoc Tukey's HSD test for comparisons of relaxation time of kidneys and livers up to 2 d post macrophage administration. ANOVA in the IRI model: livers $p = 1.8x10^{-5}$, kidneys $p = 2.2x10^{-12}$.

**Comparison**	**IRI Model**	
	**Liver**	**Kidneys**
Day 0-Baseline	0.0000638	0.0000000
Day 1-Baseline	0.0003025	0.8631656
Day 2-Baseline	0.0003586	0.8467426
Day 1-Day 0	0.9744636	0.0000000
Day 2-Day 0	0.9999998	0.0000000
Day 2-Day 1	0.9813365	0.9996660

ESM Table 3. p-values from paired t-test comparisons between the relaxation time of injured and uninjured kidneys. No statistically significant different was found in any case.

**Day**	**p-value MSCs**	**p-value RAWs**
Baseline	0.93	0.93
Day 0	0.80	0.14
Day 1	0.19	0.76
Day 2	0.29	0.66
